# Dietary Profile of Patients with Inflammatory Bowel Disease in Clinical Remission—A Preliminary Study

**DOI:** 10.3390/nu16142227

**Published:** 2024-07-11

**Authors:** Raquel Susana Torrinhas, Ilanna Marques Gomes da Rocha, Danielle Cristina Fonseca, Helena Menezes, Ana Paula Prudêncio, Bianca Depieri Balmant, Letícia Callado, Adérson Omar Mourão Cintra Damião, Natalia Queiroz, Dan L. Waitzberg

**Affiliations:** 1 Laboratory of Nutrition and Metabolic Surgery (LIM-35), Department of Gastroenterology, Faculdade de Medicina, Hospital das Clinicas HCFMUSP, Universidade de São Paulo, São Paulo 13563-120, Brazil; rtorrinhas@gmail.com (R.S.T.); daniellecf25@gmail.com (D.C.F.); menezes.helena.nutri@gmail.com (H.M.); apprudencio@gmail.com (A.P.P.); biancadepieribalmant@hotmail.com (B.D.B.); leticiacallado1@gmail.com (L.C.); dan.waitzberg@gmail.com (D.L.W.); 2 Department of Gastroenterology, Faculdade de Medicina, Hospital das Clinicas HCFMUSP, Universidade de São Paulo, São Paulo 13563-120, Brazil; aderson_damiao@uol.com.br (A.O.M.C.D.); nataliasfqueiroz@gmail.com (N.Q.)

**Keywords:** inflammatory bowel disease, Crohn’s disease, ulcerative colitis, nutrients, body composition

## Abstract

Imbalanced dietary intake is associated with the development of inflammatory bowel diseases (IBDs) and is often observed during the active phases of Crohn’s disease (CD) and ulcerative colitis (UC). Cumulative data also suggest the potential for dietary manipulation in avoiding IBD relapse. However, there is a paucity of dietary data from patients in clinical remission to guide such an approach. Our study aimed to characterize the dietary pattern and adequacy of patients with IBD in clinical remission. Data on dietary intake (three alternate 24 h food records) were collected from 40 patients with IBD (20 CD and 20 UC) and 45 gender-matched healthy controls (HC). Statistical comparisons between patients and controls employed Student’s *t*-test, Mann–Whitney U, chi-squared, and Fisher’s exact tests. The adequacy of dietary intake of IBD patients was further studied by assessing the nutrient inadequacy prevalence, estimated using the Dietary Reference Intakes (DRI) framework and the Estimated Average Requirement (EAR) parameter. We observed significant dietary imbalances among patients with IBD compared to the HC group, marked by disparities in both macronutrient and micronutrient intakes. Inadequacies with frequencies >80% were observed for the ingestion of total fiber and 13 micronutrients in IBD patients. Our preliminary findings suggest that imbalanced dietary intake is also characteristic among individuals with IBD during clinical remission, corroborating the need for dietary interventions in this population.

## 1. Introduction

Inflammatory bowel diseases (IBDs), primarily ulcerative colitis (UC) and Crohn’s disease (CD), are chronic and recurrent gastrointestinal disorders characterized by a multifaceted clinical picture with a spectrum of both intestinal and extraintestinal symptoms [1]. Epidemiological data indicate that the global prevalence of IBD has been on the rise, with an estimated six million individuals worldwide grappling with this condition [2,3]. This escalating incidence has been particularly important in the Western population, prompting extensive investigations into the role of environmental factors, including dietary choices, in the pathogenesis and prognosis of IBD [4,5].

IBD can significantly impact the physical, psychological, and social aspects of a patient’s life, particularly during the active phases of the disease. Consequently, the primary therapeutic goal is to achieve and maintain clinical, laboratory, and endoscopic remission [6]. However, despite being fundamental in the management of CD and UC over the last few decades, pharmacotherapy does not always produce the desired outcomes and is often associated with an increased likelihood of adverse repercussions in affected patients [7].

Nutritional strategies, whether as adjunctive or primary interventions, have garnered significant attention in the care of IBD patients [8]. Various factors render these patients vulnerable to nutritional risks throughout different stages of the disease, including inadequate dietary intake, nutrient malabsorption due to enteral losses, elevated basal energy expenditure, and the impact of pharmacological treatments [9]. This inherent susceptibility to nutritional deficiencies suggests a potential role of nutrition in both the pathogenesis and therapeutic management of IBD [10].

One of the most critical determinants of IBD-related malnutrition is the inadequate intake of both macronutrients and micronutrients [11]. This dietary deficiency can be driven by various factors, including restricted diets, malabsorption, and the avoidance of certain foods due to disease-related symptoms. Appropriate nutritional therapy has been shown to improve nutritional status, enhance postoperative healing, reduce hospitalization time, and improve prognosis [12,13,14].

Recognizing the importance of diet in the treatment of IBD, our study investigated dietary inadequacies in patients with IBD who are in clinical remission, comparing them to a healthy control group. Understanding the dietary patterns and nutritional deficiencies in IBD patients during remission phases can be useful for designing dietary strategies aiming to improve long-term disease management and quality of life.

## 2. Materials and Methods

### 2.1. Ethical Issues

This study adhered to the ethical principles of the Declaration of Helsinki. The research protocol was registered at Plataforma Brazil and obtained approval from the local Ethics Committee (CAAE 01713018.0.0000.0068. Protocol number: 4.082.713). All participants involved in the study provided informed written consent, ensuring that their rights and privacy were respected throughout the research process.

### 2.2. Selection of Participants

#### 2.2.1. Patients (IBD Group)

Patients with IBD were recruited from the Inflammatory Bowel Diseases Ambulatory at the Hospital das Clinicas da Faculdade de Medicina da Universidade de Sao Paulo, Department of Gastroenterology, São Paulo, Brazil. The inclusion criteria were as follows: individuals of both genders with confirmed diagnoses of UC (n = 20) or CD (n = 20) who were in sustained clinical remission for at least 1 year and fully adhered to clinical treatment with regular use of prescribed medications. The IBD diagnosis was made based on the criteria established by the European Crohn and Colitis Organization (ECCO) [15,16], and clinical remission was defined using specific criteria: a Harvey–Bradshaw index score of ≤4 for CD [17] and a partial Mayo score (Mayo score without the endoscopy component) of ≤2 points for UC, without any individual score exceeding 1 point [18]. Exclusion criteria were as follows: confirmed diagnosis of celiac disease, irritable bowel syndrome, small intestinal bacterial overgrowth, previous gastrointestinal surgery, use of special diets (e.g., ketogenic diet and intermittent fasting), enteral nutrition, and extreme overall calorie intake (<800 or >4000 kcal/day for men; <500 or >3500 kcal/day for women).

#### 2.2.2. Healthy Controls (HC Group)

To establish a suitable control group, healthy individuals (n = 45) matched by gender with IBD patients were recruited from the local area. Extensive screening ensured these individuals met specific criteria designed to minimize confounding variables and create a representative control group. Inclusion criteria were the absence of any chronic or acute disease, no regular use of medication, no dietary restrictions or unusual dietary habits, no history of gastrointestinal surgery (except appendectomy), a body mass index (BMI) of ≤30 kg/m^2^, absence of drug or alcohol abuse, no cognitive deficits, and not being pregnant or breastfeeding.

### 2.3. Demographic and Clinical Variables

Demographic and clinical information, including age, gender, declared ethnicity, smoking, anthropometry, percentage of fat and lean mass, stool frequency, and stool consistency, were collected from both the IBD and HC groups. Anthropometric examination was performed in the morning in the fasting state to record the following variables: body weight (kg), measured using an electronic platform scale (Life Measurement Instruments, Concord, CA, USA) with subjects wearing light clothing and no shoes; height (m), measured using a stadiometer (Sanny; American Medical do Brasil, São Paulo, Brazil) with subjects standing barefoot, heels together, spine erect, and arms extended alongside the body; and BMI, calculated as weight divided by height squared (kg/m^2^) and classified as underweight (<18.5 kg/m^2^), normal weight (18.5–24.9 kg/m^2^), overweight (25–29.9 kg/m^2^), and obesity (>30 kg/m^2^), according to the World Health Organization (WHO) definition [19]. Body composition was measured using bioelectric impedance analysis (QuadScan4000; Bodystat, Douglas, Isle of Man, British Isles). Measurements were taken with subjects in a fasting state, after urination, and without jewelry or wristwatches to record fat mass and muscle mass percentual variables. The stool consistency was classified according to the Bristol stool scale as follows: tendency toward constipation (types 1–2), ideal stool (types 3–4), and tendency toward diarrhea (types 5–6).

### 2.4. Food Consumption Pattern

The dietary consumption of both IBD and HC groups was assessed through 24 h dietary recalls (R24) conducted on three non-consecutive days (two weekdays and one weekend day), with no prior nutritional guidance given, ensuring the data accurately reflected their usual dietary habits. Trained researchers administered the dietary recalls using a structured and semi-directed interview format, which included detailed inquiries about food consumption, preparations, and meal timings. Participants provided specific details such as household measurements, ingredients, cooking methods, brand names of processed foods, and additional information regarding added salt, sugar, oils, sauces, diet and light products, dietary supplements, and multivitamins [20]. 

The dietary consumption records obtained from the R24 applications were tabulated using Microsoft Excel^®^ (Microsoft, Office Suite, 2013), with the conversion and standardization of the reported household measurements into grams based on Brazilian reference tables [21,22]. In cases where information was absent from these tables, technical sheets for preparations were created detailing the preparation’s name, constituent ingredients, quantities used, preparation methods, and values in grams equivalent to the household measurements reported by the participant or as standardized [23]. For processed foods, product labels were consulted on the supplying industry’s website. 

The dietary data were then analyzed using Easy Diet^®^ software (Version 1) to calculate dietary energy value and nutrient content. The software utilized the Brazilian Table of Food Composition [24] and the United States Department of Agriculture [25] for nutritional calculations. After verifying the nutrient values obtained through EasyDiet^®^, the data were processed using the Multiple Source Method statistical program to estimate individual habitual dietary consumption, considering adjustment variables [26].

### 2.5. Food Consumption Adequacy

The prevalence of nutrient inadequacy was estimated using the Dietary Reference Intakes (DRI) framework, specifically employing the Estimated Average Requirement (EAR) parameter [27]. Nutrient adequacy was defined as the consumption of values exceeding the EAR, which represents the median requirement of a nutrient for a group of individuals of the same sex and life stage. For nutrients lacking EAR values, adequate intake (AI) values served as the reference parameter. The prevalence of inadequacy for each nutrient was calculated based on the percentage of individuals whose habitual intake was below the EAR or AI appropriate for their gender and age group.

### 2.6. Statistical Analysis

Descriptive analysis provided frequencies (percent) for categorical variables and mean ± standard deviation, or median (with interquartile range) for continuous variables. The normality of continuous variables was assessed using the Shapiro–Wilk test. Comparisons between groups were performed using the Student’s *t*-test and Mann–Whitney U test for continuous variables, and the chi-squared and Fisher’s exact tests for categorical variables. 

## 3. Results

### 3.1. Descriptive Data 

The comparison of demographic and clinical data between the IBD and HC groups is presented in Table 1. Compared to the HC group, patients in the IBD group exhibited a higher average age and BMI, a higher prevalence of overweight/obesity, a greater tendency toward diarrhea, a lower prevalence of ideal stools, and poor body composition, characterized by a higher percentage of fat mass and a lower percentage of lean mass. On the other hand, the comparison within the IBD group showed no significant differences in the investigated clinical and demographic variables, indicating homogeneity between the CD and UC subgroups (*p* > 0.050). Additionally, no significant differences were found between disease location in UC and disease location and behavior in CD (*p* > 0.050). 

### 3.2. Dietary Patterns

The comparison of dietary intake, including macro and micronutrient consumption, between the IBD and HC groups is presented in Table 2. Among the macronutrients, patients with IBD exhibited a higher carbohydrate intake while consuming lower amounts of proteins and total fat compared to the HC group. Additionally, they showed significantly lower intake of several micronutrients, including calcium, phosphorus, copper, potassium, zinc, and folic acid. On the other hand, the comparison within the IBD group showed a similar dietary pattern between the CD and UC subgroups for all macronutrients and micronutrients analyzed, except for higher phosphorus consumption in the CD subgroup (477.6 ± 106.2 vs. 407.0 ± 157.5; *p* = 0.040).

### 3.3. Dietary Adequacy

Table 3 shows the daily nutritional recommendations for macronutrients and micronutrients according to the Dietary Reference Intakes (DRI), the average dietary intake of patients from the IBD group, and the prevalence of inadequate intake based on reference parameters for sex and age group. Female patients showed prevalence rates of inadequacy greater than 80% for the consumption of fiber, calcium, selenium, vitamin B6, phosphorus, folate, vitamin B3, vitamin B1, vitamin E, and vitamin D in at least one of the age groups. Except for vitamin B1 and phosphorus, male patients had prevalence rates of inadequacy greater than 80% for these same nutrients, as well as for magnesium, manganese, and vitamin A in at least one of the age groups. It is also noteworthy that there was 100% inadequacy in the daily intake of some micronutrients, such as vitamins B6, folate, vitamin D, and vitamin E, for specific age groups, particularly those aged over 51 years.

## 4. Discussion

Malnutrition is a common concern in active IBD, primarily resulting from poor nutrient absorption and chronic inflammation of the gastrointestinal tract [19]. Additionally, IBD patients often self-impose dietary restrictions, avoiding high-fiber, dairy, fatty, and spicy foods to control symptoms and prevent clinical relapse during remission [28]. Recent data indicate that the prevalence of malnutrition in hospitalized IBD patients ranges from 20% to 85% [12].

Paradoxically, patients with IBD can exhibit a high BMI, possibly due to corticosteroid use and a sedentary lifestyle [29,30]. Specifically, in Western countries, more than half of IBD patients in clinical remission are obese or overweight [31]. Furthermore, up to 60% of IBD patients have decreased muscle mass compared to healthy subjects, with 52% of CD patients and 37% of UC patients being sarcopenic [32].

The altered body composition in IBD patients, mainly low lean muscle mass, can adversely affect disease progression, treatment response, surgical outcomes, and overall quality of life [33,34]. In our study, IBD patients in clinical remission exhibited higher BMI and obesity prevalence, with an increased percentage of body fat and a decreased percentage of lean body mass compared to healthy controls. Our data align with the nutritional and body composition phenotype reported in IBD patients in clinical remission, potentially contributing to impaired outcomes.

Our findings in body composition also corroborate the need to extend the nutritional assessment of IBD patients beyond traditional anthropometric analyses and BMI calculations [34]. Relying solely on BMI can mask nutritional inadequacies, such as the reduction in lean mass being offset by an increase in fat mass [35]. Consequently, evaluating muscle mass in IBD patients is increasingly becoming a standard component of clinical practice. 

The reduction in muscle mass in IBD can be attributed to factors such as diminished oral intake, chronic inflammation, and short bowel syndrome [36]. Our study explored oral intake, identifying several differences in macro and micronutrient ingestion compared to gender-matched healthy controls. Notably, while most differing macronutrient levels were within the daily recommended intake (DRI), the opposite was observed for the differing micronutrients, which were often below the recommended levels [27].

Curiously, the differences we observed in macronutrient consumption in IBD patients were marked by a higher intake of carbohydrates compared to controls. In a previous large study, a very high carbohydrate diet was associated with metabolic risk factors, as well as low energy and saturated fat intake [37]. Similarly, in our study, IBD patients exhibited higher carbohydrate intake along with lower energy (non-significant), protein, and fat (including saturated fat; *p* ≤ 0.050) consumption compared to healthy controls. Since carbohydrate intake was within DRI recommendations, our findings suggest that even a slight increase in carbohydrates can alter macronutrient balance, potentially impairing metabolism.

In IBD, reduced caloric–protein consumption, especially in the elderly, exposes patients to a higher risk of muscle tissue catabolism and sarcopenia [38]. During the active phase of the disease, macronutrient deficits are often reported and attributed to persistent gastrointestinal symptoms such as nausea, vomiting, and abdominal pain, as well as the fear of eating. However, adequate dietary consumption of these nutrients during clinical remission is expected, as observed in our study, for all macronutrients except fiber [39]. 

Notably, fiber consumption was below recommendations in both patients and controls, aligning with observations of fiber depletion in general populations adopting a Western diet [40]. In IBD patients, dietary fiber plays a crucial role in intestinal health; its fermentation in the large intestine regulates intestinal lumen pH, promotes the synthesis of vitamins (especially vitamin K and B-complex vitamins), and stimulates the production of short-chain fatty acids (SCFAs) [41,42,43,44]. Diets low in fiber, on the other hand, are associated with a reduction in fermentative species and lower SCFA production, which serves as a vital energy source for colonocytes [41].

In our study, altered micronutrient intake in IBD patients compared to healthy controls included a higher intake of vitamin B6 and a lower intake of calcium, magnesium, phosphorus, and copper, with compliance to DRI recommendations below 20% in at least one gender or age range. Additionally, IBD patients exhibited similar intakes to healthy controls for several micronutrients, including selenium, manganese, folate, and vitamins A, D, E, B1, and B3, with compliance to DRI recommendations also below 20% in at least one gender or age range. These observations underscore a potential poor compliance in micronutrient intake among healthy individuals as well.

Biochemical deficiencies for several micronutrients have been previously reported in IBD patients during clinical remission, including vitamin D (29%), zinc (16%), vitamin B6 (14%), vitamin C (13%), vitamin B12 (11%), folate (8%), ferritin (9%), copper (4%), magnesium (4%), and selenium (3%). Zinc deficiency, in particular, was predictive of a shorter time to subsequent relapse, especially in the Crohn’s disease subgroup [45]. In our study, we observed a depleted intake of 70% of these dietary components (excluding zinc), suggesting that dietary consumption may be the primary factor responsible for micronutrient depletion in IBD patients in clinical remission.

A recent systematic review showed that Crohn’s disease (CD) patients in clinical remission are likely to have a range of micronutrient deficiencies, with strong evidence for vitamin D and B12 depletion [46]. In our study, we found that 100% of IBD patients had inadequate vitamin D intake, regardless of gender or age range, while vitamin B12 consumption was 79.9% below recommendations for women and 22.0% for men. These findings reinforce the potential impact of dietary intake on micronutrient depletion in IBD patients during clinical remission and suggest that gender may influence this process. 

The ingestion of vitamin E and folate was also 100% depleted/deficient in our population of IBD patients. Vitamin E has shown the potential to ameliorate intestinal inflammation and improve mucosal barrier function in experimental IBD models, with supplementation in CD patients reducing oxidative stress [47,48]. Folate deficiency affects approximately 30% of CD patients and 10% of UC patients, primarily due to malabsorption and low intake, and is associated with anemia. Folate is essential for nucleotide synthesis and plays a role in regulating inflammation, as it is involved in the function of regulatory T cells [49].

Taken together, our observations highlight a prevalent dietary micronutrient inadequacy in IBD patients in clinical remission and reinforce the need for dietary interventions to ensure adequate intake. However, specific nutritional guidelines for this subset of the IBD population are lacking. Dietary strategies for individuals with IBD in clinical remission can include specific diets, such as the low-FODMAP diet, to avoid inflammation and symptoms [50]. Our data also support the inclusion of nutritional supplementation with vitamins and minerals to address deficiencies.

Our study has several limitations to highlight. The sample size may not fully represent the diverse IBD population, and recruitment from a specific geographical area and clinical setting introduces potential selection bias. Consequently, data generalization and interpretation should be approached with caution, given their preliminary nature. Additionally, reliance on self-reported dietary intake data poses an inherent risk of recall bias. The cross-sectional design restricts the establishment of causal relationships between dietary habits, nutritional status, and clinical outcomes. Further research with longitudinal designs and larger, more diverse samples is required to address these limitations and enhance the validity of our findings.

## 5. Conclusions

In conclusion, our preliminary findings suggest that the dietary profile of IBD patients has significant micronutrient deficiencies even during clinical remission. This supports the need for ongoing nutritional monitoring and targeted interventions to address these deficiencies and potentially improve patient outcomes.

## Figures and Tables

**Table 1:** Comparison of demographic and clinical data between the healthy control group and patients with inflammatory bowel disease in clinical remission.

Variable	HC (n = 45)	IBD (n = 40)	*p*-Value
Age (years)	38.3 ± 15.1	44.8 ± 14.7	**0.** **013 ^a^**
Caucasian	35 (77.8%)	23 (57.0%)	0.062 ^b^
Not-Caucasian	10 (22.2%)	17 (43.0%)	
Male	24 (53.3%)	16 (40.0%)	0.278 ^b^
Female	21 (46.7%)	24 (60.0%)	
Weight (kg)	68.2 ± 13.5	70.4 ± 14.2	0.440 ^a^
Height (cm)	1.7 ± 0.1	1.7 ± 0.1	0.150 ^a^
BMI (kg/m^2^)	23.7 ± 2.9	25.5 ± 4.1	**0.** **048 ^a^**
Underweight	2 (4.4%)	1 (2.5%)	**0.** **033 ^b^**
Normal	25 (55.6%)	18 (45.0%)	
Overweight	18 (21. 2%)	14 (35.0%)	
Obesity	0 (0.0%)	7 (17.5%)	
Fat mass (%)	27.2 ± 7.4	33.1 ± 8.1	**0.** **001 ^a^**
Muscle mass (%)	72.78 ± 7.4	66.9 ± 8.1	**0.** **001 ^a^**
Tendency toward constipation	3 (6.8%)	2 (5.0%)	**0.022 ^b^**
Ideal stool	38 (86.4%)	25 (62.5%)	
Tendency toward diarrhea	3 (6.8%)	13 (32.5%)	
Non-smoking	45 (00.0%)	37 (92.5%)	0.100 ^c^
Smoking	0 (0.0%)	3 (7.5%)	

Legends: HC: Healthy control; IBD: Inflammatory bowel disease; BMI: Body mass index. Data are expressed as mean ± standard deviation or number of events (percentage) and compared by ^a^ Mann–Whitney test; ^b^ chi-square test; ^c^ Fisher’s exact test. Significant differences are highlighted in bold.

**Table 2:** Comparison of habitual macronutrient and micronutrient intake between patients with inflammatory bowel disease and healthy control group in clinical remission.

Energy/Nutrient	HC (n = 45)	IBD (n = 40)	*p*-Value
Energy (kcal)	1893.5 ± 556.7	1738.8 ± 385.2	0.3877
Carbohydrates (g)	214.9 ± 75.9	232.6 ± 51.8	**0.0330**
Fiber (g)	20.4 ± 7.3	20.6 ± 7.3	0.6127
Protein (g)	91.4 ± 26.8	70.7 ± 16.7	**0.0001**
Fat (g)	73.3 ± 22.7	59.4 ± 14.3	**0.0055**
Saturated fat (g)	25.8 ± 7.2	21.1 ± 5.8	**0.0023**
Monounsaturated fat (g)	21.8 ± 5.9	17.3 ± 3.8	**0.0003**
Polyunsaturated fat (g)	16.5 ± 5.9	13.6 ± 4.9	**0.0239**
Cholesterol (g)	354.3 ± 185.5	245.6 ± 69.2	**0.0005**
Sodium (mg)	3485.2 ± 966.7	3099.2 ± 947.9	0.0563
Calcium (g)	696.7 ± 202.9	561.2 ± 200.9	**0.0003**
Iron (g)	9.3 ± 2.8	8.3 ± 2.1	0.2730
Magnesium (mg)	257.1 ± 69.5	218.1 ± 53.4	**0.0180**
Selenium (µg)	23.7 ± 8.4	24.8 ± 7.7	0.1926
Vitamin C (mg)	136.3 ± 71.4	104.6 ± 42.8	0.0651
Vitamin B1 (mg)	1.0 ± 0.4	0.8 ± 0.2	0.0943
Vitamin B6 (mg)	0.6 ± 0.2	0.7 ± 0.2	**0.0135**
Phosphorus (mg)	538.7 ± 173.9	442. 3 ± 137.3	**0.0032**
Copper (mg)	3.9 ± 7.2	0.7 ± 0.7	**0.0000**
Manganese (mg)	0.9 ± 0.4	1.2 ± 1.3	0.5493
Potassium (mg)	2502.9 ± 804.4	2017.8 ± 409.0	**0.0057**
Zinc (mg)	10.1 ± 2.4	8.4 ± 2.3	**0.0022**
Vitamin B3 (mg)	5.8 ± 2.6	4.81 ± 2.3	0.0671
Folic Acid (µg)	107.4 ± 37.9	87.5 ± 28.8	**0.0071**
Vitamin E (mg)	1.9 ± 0.4	1.8 ± 0.6	0.0917
Vitamin B12 (µg)	2.1 ± 1.2	1.7 ± 0.7	0.2469
Vitamin D (µg)	1.8 ± 0.9	1.9 ± 0.7	0.3009
Vitamin A (µg)	354.0 ± 222.2	349.3 ± 249.2	0.7181

Legends: HC: healthy control group; IBD: inflammatory bowel disease. Data expressed as mean ± standard deviation and compared by Wilcoxon (Mann–Whitney) test. Significant differences are highlighted in bold.

**Table 3:** Nutritional recommendations, habitual consumption of macronutrients and micronutrients, and prevalence of inadequate intake by sex and age group in patients with inflammatory bowel diseases in clinical remission.

	Female (n = 24)			Male (n = 16)		
Nutrients	EAR	Mean (SD)	%IN	EAR	Mean (SD)	%IN
Carbohydrate (g)						
19 to 70 years or more	130.0	219.8 (46.8)	2.7	130.0	251.8 (54.4)	1.3
Fiber (g)						
19–50 years	25.0	18.7 (6.7)	**82.** **4**	38.0	21.8 (7.4)	**98.** **5**
51 years or more	21.0	21.0 (8.8)	51.6	30.0	22.6 (6.7)	**86.** **4**
Protein (g)						
19 years or more	46.0	62.4 (14.6)	12.9	56.0	81.5 (13.0)	2.5
Sodium (mg)						
19 to 71 years or more	2300.0	2600.3 (667.3)	32.6	2300.0	3487.7(810.8)	2.8
Calcium (mg)						
19 to 45 years	800.0	514.2 (105.4)	**99.** **7**	800.0	730.4 (279.9)	59.5
46 years or more	1000.0	484.0 (122.9)	**100** **.0**	1000.0	527.8 (194.7)	**99.** **2**
Iron (mg)						
19 to 45 years	8.1	7.9 (2.2)	53.6	6	9.8 (1.7)	1.5
46 years or more	5.0	7.5 (1.5)	5.5	6	8.3 (2.2)	14.9
Magnesium (μg)						
19 to 30 years	255.0	214.5 (68.8)	71.9	330.0	203.1 (62.5)	**97.** **8**
31 years or more	265.0	209.4 (53.9)	79.7	350.0	238.8 (47.9)	**99.0**
Selenium (μg)						
19 to 71 years or more	45.0	25.8 (8.7)	**98.** **6**	45.0	23.4 (5.8)	**100.0**
Vitamin C (mg)						
19 to 71 years	60.0	108.3 (49.3)	16.3	75.0	99.1 (31.3)	22.4
Vitamin B1 (mg)						
19 to 71 years	0.9	0.7 (0.2)	**81.** **1**	0.9	0.9 (0.2)	52.4
Vitamin B6 (mg)						
19 to 50 years	1.1	0.8 (0.1)	**98.** **5**	1.1	0.8 (0.1)	**98.** **1**
51 years or more	1.3	0.5 (0.1)	**100** **.0**	1.4	0.7 (0.1)	**100** **.0**
Phosphorus (mg)						
19 to 71 years	580.0	403.3 (121.3)	**92.** **6**	580.0	500.8 (142.7)	70.9
Copper (mg)						
19 to 71 years	700.0	797.9 (872.8)	45.0	700.0	606.9 (275.0)	62.9
Manganese (mg)						
19–71 years	1.8	1.4 (1.5)	61.0	2.3	0.9 (0.6)	**99.** **1**
Zinc (mg)						
19 to 71 years	6.8	7.1 (1.5)	41.3	9.4	10.28 (1.92)	29.1
Vitamin B3 (mg)						
19 to 71 years	11.0	4.6 (2.5)	**99.** **4**	12.0	5.2 (2.0)	**100.0**
Folate (µg)						
19 to 71 years	320.0	94.0 (28.2)	**100** **.0**	320.0	77.9 (27.6)	**100** **.0**
Vitamin E (mg)						
19 to 71 years	12.0	1.8 (0.6)	**100** **.0**	12.0	1.7 (0.6)	**100** **.0**
Vitamin B12 (μg)						
19 to 71 years	2.0	1.5 (0.5)	79.9	2.0	2.1 (0.8)	22.0
Vitamin D (μg)						
19 to 50 years	5.0	2.1 (0.8)	**100.0**	5.0	2.3 (0.5)	**100** **.0**
51 to 70 years	10.0	1.5 (0.3)	**100** **.0**	10.0	1.6 (0.9)	**100** **.0**
Vitamin A (µg)						
19 to 71 years	500.0	352.8 (213.3)	75.5	625.0	344.1 (302.7)	**82.** **2**

Legend: SD: Standard deviation; %IN: Percentage of inadequacy; EAR: Estimated average requirement. Inadequacies greater than 80% are highlighted in bold.

## Data Availability

Data are contained within the article.

## References

[1] Ananthakrishnan A.N., Bernstein C.N., Iliopoulos D., Macpherson A., Neurath M.F., Ali R.A.R., Vavricka S.R., Fiocchi C. (2018). Environmental triggers in IBD: A review of progress and evidence. Nat. Rev. Gastroenterol. Hepatol..

[2] AGA Patient Information Section (2017). Inflammatory Bowel Disease. Clin. Gastroenterol. Hepatol..

[3] Kudelka M.R., Stowell S.R., Cummings R.D., Neish A.S. (2020). Intestinal epithelial glycosylation in homeostasis and gut microbiota interactions in IBD. Nat. Rev. Gastroenterol. Hepatol..

[4] Park J., Cheon J.H. (2021). Incidence and Prevalence of Inflammatory Bowel Disease across Asia. Yonsei Med. J..

[5] Harris K.G., Chang E.B. (2018). The intestinal microbiota in the pathogenesis of inflammatory bowel diseases: New insights into complex disease. Clin. Sci..

[6] GBD 2017 Inflammatory Bowel Disease Collaborators (2020). The global, regional, and national burden of inflammatory bowel disease in 195 countries and territories, 1990–2017: A systematic analysis for the Global Burden of Disease Study 2017. Lancet Gastroenterol. Hepatol..

[7] Seyedian S.S., Nokhostin F., Malamir M.D. (2019). A review of the diagnosis, prevention, and treatment methods of inflammatory bowel disease. J. Med. Life.

[8] Guo X., Huang C., Xu J., Xu H., Liu L., Zhao H., Wang J., Huang W., Peng W., Chen Y. (2022). Gut Microbiota Is a Potential Biomarker in Inflammatory Bowel Disease. Front. Nutr..

[9] Scaldaferri F., Pizzoferrato M., Lopetuso L.R., Musca T., Ingravalle F., Sicignano L.L., Gasbarrini A. (2017). Nutrition and IBD: Malnutrition and/or Sarcopenia? A Practical Guide. Gastroenterol. Res. Pract..

[10] Nardone O.M., de Sire R., Petito V., Testa A., Villani G., Scaldaferri F., Castiglione F. (2021). Inflammatory Bowel Diseases and Sarcopenia: The Role of Inflammation and Gut Microbiota in the Development of Muscle Failure. Front. Immunol..

[11] Cioffi I., Imperatore N., Di Vincenzo O., Pagano M.C., Santarpia L., Pellegrini L., Pasanisi F. (2020). Evaluation of Nutritional Adequacy in Adult Patients With Crohn’s Disease: A Cross-Sectional Study. Eur. J. Nutr..

[12] Massironi S., Viganò C., Palermo A., Pirola L., Mulinacci G., Allocca M., Peyrin-Biroulet L., Danese S. (2023). Inflammation and malnutrition in inflammatory bowel disease. Lancet Gastroenterol Hepatol..

[13] Yamashiro Y. (2018). Gut Microbiota in Health and Disease. Ann. Nutr. Metab..

[14] Khalili H., Chan S.S.M., Lochhead P., Ananthakrishnan A.N., Hart A.R., Chan A.T. (2018). The role of diet in the aetiopathogenesis of inflammatory bowel disease. Nat. Rev. Gastroenterol. Hepatol..

[15] Magro F., Gionchetti P., Eliakim R., Ardizzone S., Armuzzi A., Barreiro-de Acosta M., Burisch J., Gecse K.B., Hart A.L., European Crohn’s and Colitis Organisation [ECCO] (2017). Third European evi-dence-based consensus on diagnosis and management of ulcerative colitis. Part 1: Definitions, diagnosis, extra-intestinal manifestations, pregnancy, cancer surveillance, surgery, and ileo-anal pouch disorders. J. Crohn’s Colitis.

[16] Gomollón F., Dignass A., Annese V., Tilg H., Van Assche G., Lindsay J.O., Peyrin-Biroulet L., Cullen G.J., Daperno M., Kucharzik T. (2017). 3rd European evidence-based consensus on the diagnosis and management of Crohn’s disease 2016: Part 1:Diagnosis and medical management. J. Crohn’s Colitis.

[17] Harvey R.F., Bradshaw M.J. (1980). Measuring Crohn’s disease activity. Lancet.

[18] Sandborn W.J., van Assche G., Reinisch W., Colombel J., D’haens G., Wolf D.C., Kron M., Tighe M.B., Lazar A., Thakkar R.B. (2012). Adalimumab induces and maintains clinical remission in patients with moderate-to-severe ulcerative colitis. Gastroenterology.

[19] Balestrieri P., Ribolsi M., Guarino M.P.L., Emerenziani S., Altomare A., Cicala M. (2020). Nutritional Aspects in Inflammatory Bowel Diseases. Nutrients.

[20] Fisberg R.M., Marchioni D.M.L., Colucci A.C.A. (2009). Avaliação do consumo alimentar e da ingestão de nutrientes na prática clínica. Arq. Bras. De Endocrinol. Metabol..

[21] Brazilian Institute of Geography and Statistics (IBGE) (2011). Table of Measures Referred to the Foods Consumed in Brazil.

[22] Giuntini E.B., Lajolo F.M., Menezes E.W. (2006). Tabela Brasileira de Composição de Alimentos TBCA-USP (Versões 3 e 4) no contexto internacional. Arch. Latinoam. Nutr..

[23] Pinheiro A.B.V., Lacerda E.M.D.A., Benzecry E.H., Gomes M.C.D.S., Costa V.M.D. (2008). Tabela Para Avaliação de Consumo Alimentar Em Medidas Caseiras.

[24] NEPA—UNICAMP (2004). Tabela Brasileira de Composição de Alimentos (TACO).

[25] United States Department of Agriculture (USDA) (2012). National Nutrient Database for Standard Reference.

[26] The Multiple Source Method (MSM) (2011). Department of Epidemiology of the German Institute of Human Nutrition Potsdam-Rehbrücke (DiFE). https://nugo.dife.de/msm.

[27] Padovani R.M., Amaya-Farfan J., Colugnati F.A.B., Domene S.M.A. (2006). Dietary reference intakes: Aplicabilidade das tabelas em estudos nutricionais. Rev. Nutr..

[28] Cohen A.B., Lee D., Long M.D., Kappelman M.D., Martin C.F., Sandler R.S., Lewis J.D. (2013). Dietary patterns and self-reported associations of diet with symptoms of inflammatory bowel disease. Dig Dis. Sci..

[29] Kaazan P., Tan Z., Maiyani P., Mickenbecker M., Edwards S., McIvor C., Andrews J.M. (2022). Weight and BMI Patterns in a Biologicals-Treated IBD Cohort. Dig Dis. Sci..

[30] Mareschal J., Douissard J., Genton L. (2022). Physical activity in inflammatory bowel disease: Benefits, challenges and perspectives. Curr. Opin. Clin. Nutr. Metab. Care.

[31] Schreiner P., Martinho-Grueber M., Studerus D., Vavricka S.R., Tilg H., Biedermann L. (2020). on behalf of Swiss IBD net, an official working group of the Swiss Society of Gastroenterology. Nutrition in Inflammatory Bowel Disease. Digestion.

[32] Dhaliwal A., Quinlan J.I., Overthrow K., Greig C., Lord J.M., Armstrong M.J., Cooper S.C. (2021). Sarcopenia in Inflammatory Bowel Disease: A Narrative Overview. Nutrients.

[33] Potcovaru C.G., Filip P.V., Neagu O.M., Diaconu L.S., Salmen T., Cinteză D., Pantea Stoian A., Bobirca F., Berteanu M., Pop C. (2023). Diagnostic Criteria and Prognostic Relevance of Sarcopenia in Patients with Inflammatory Bowel Disease-A Systematic Review. J. Clin. Med..

[34] Ding N.S., Tassone D., Al Bakir I., Wu K., Thompson A.J., Connell W.R., Malietzis G., Lung P., Singh S., Choi C.R. (2022). Systematic Review: The Impact and Importance of Body Composition in Inflammatory Bowel Disease. J. Crohns Colitis.

[35] Bryant R.V., Trott M.J., Bartholomeusz F.D., Andrews J.M. (2013). Systematic review: Body composition in adults with inflammatory bowel disease. Aliment. Pharmacol. Ther..

[36] Ryan E., McNicholas D., Creavin B., Kelly M.E., Walsh T., Beddy D. (2019). Sarcopenia and Inflammatory Bowel Disease: A Systematic Review. Inflamm. Bowel Dis..

[37] Lee Y.J., Song S., Song Y. (2018). High-Carbohydrate Diets and Food Patterns and Their Associations with Metabolic Disease in the Korean Population. Yonsei Med. J..

[38] Yueying C., Yu Fan W., Jun S. (2020). Anemia and iron deficiency in Crohn’s disease. Expert Rev. Gastroenterol. Hepatol..

[39] Massironi S., Cavalcoli F., Rausa E., Invernizzi P., Braga M., Vecchi M. (2020). Understanding short bowel syndrome: Current status and future perspectives. Dig Liver Dis..

[40] FAO/OMS Fiber Dietary Recommendation. Disponível Em. https://www.who.int/dietphysicalactivity/fruit/en/.

[41] Statovci D., Aguilera M., MacSharry J., Melgar S. (2017). The Impact of Western Diet and Nutrients on the Microbiota and Immune Response at Mucosal Interfaces. Front. Immunol..

[42] Rowland I., Gibson G., Heinken A., Scott K., Swann J., Thiele I., Tuohy K. (2018). Gut microbiota functions: Metabolism of nutrients and other food components. Eur. J. Nutr..

[43] Precup G., Vodnar D.C. (2019). Gut Prevotella as a possible biomarker of diet and its eubiotic versus dysbiotic roles: A comprehensive literature review. Br. J. Nutr..

[44] Cronin P., Joyce S.A., O’Toole P.W., O’Connor E.M. (2021). Dietary Fibre Modulates the Gut Microbiota. Nutrients.

[45] MacMaster M.J., Damianopoulou S., Thomson C., Talwar D., Stefanowicz F., Catchpole A., Gerasimidis K., Gaya D.R. (2021). A prospective analysis of micronutrient status in quiescent inflammatory bowel disease. Clin. Nutr..

[46] McDonnell M., Sartain S., Westoby C., Katarachia V., Wootton S.A., Cummings J.R.F. (2023). Micronutrient Status in Adult Crohn’s Disease during Clinical Remission: A Systematic Review. Nutrients.

[47] Liu K.Y., Nakatsu C.H., Jones-Hall Y., Kozik A., Jiang Q. (2021). Vitamin e alpha- and gam-ma-tocopherol mitigate colitis, protect intestinal barrier function and modulate the gut microbiota in mice. Free Radic Biol. Med..

[48] Aghdassi E., Wendland B.E., Steinhart A.H., Wolman S.L., Jeejeebhoy K., Allard J.P. (2003). Antioxidant vitamin supplementa-tion in crohn’s disease decreases oxidative stress. a randomized controlled trial. Am. J. Gastroenterol..

[49] Yamaguchi T., Hirota K., Nagahama K., Ohkawa K., Takahashi T., Nomura T., Sakaguchi S. (2007). Control of immune responses by antigen-specific regulatory T cells expressing the folate receptor. Immunity.

[50] Shafiee N.H., Manaf Z.A., Mokhtar N.M., Raja Ali R.A. (2021). Anti-inflammatory diet and inflammatory bowel disease: What clinicians and patients should know?. Intest. Res..

